# Secondary attack rates and determinants of Severe Acute Respiratory Syndrome Coronavirus 2 (SARS-CoV-2) household transmission in Pakistan: A case-ascertained prospective, longitudinal study

**DOI:** 10.1016/j.jiph.2024.03.024

**Published:** 2024-05

**Authors:** Muhammad Imran Nisar, Nadia Ansari, Mashal Amin, Farah Khalid, Shahira Shahid, Marvi Mahesar, Maryam Mansoor, Muhammad Farrukh Qazi, Aneeta Hotwani, Najeeb Rehman, Arslan Ashraf, Zahoor Ahmed, Ashfaque Ahmed, Arslan Memon, Fyezah Jehan

**Affiliations:** a Department of Pediatrics and Child Health, Aga Khan University, Karachi, Pakistan; b Health Department, Government of Sindh, Karachi, Pakistan

**Keywords:** SARS-CoV-2, COVID-19, Transmission dynamics, Secondary attack rate, Pakistan

## Abstract

**Background:**

Households are considered ideal settings for studying the transmission dynamics of an infectious disease.

**Methods:**

A prospective study was conducted, based on the World Health Organization FFX protocol from October 2020 to January,2021. Household contacts of laboratory-confirmed index cases were followed up for their symptomatic history, nasal swabs for RT-PCR,and blood samples for anti-SARS CoV-2 antibodies were collected at enrollment and days 7, 14 and 28. We estimated secondary attack rate (SAR), effective household case cluster size and determinants of secondary infection among susceptible household contacts using multivariable logistic regression.

**Results:**

We enrolled 77 index cases and their 543 contacts. Out of these, 252 contacts were susceptible at the time of enrollment. There were 77 household clusters, out of which, transmission took place in 20 (25.9%) giving rise to 34 cases. The acquired secondary attack rate (SAR) was 14.0% (95% CI 9.0–18.0). The effective household case cluster size was 0.46 (95%CI 0.33,0.56). Reported symptoms of nausea and vomiting (aOR, 7.9; 95% CI, 1.4–45.5) and fatigue (aOR, 9.3; 95% CI, 3.8–22.7) were associated with SARS-CoV-2 transmission.

**Conclusions:**

We observed a low SARS-CoV-2 secondary attack rate in the backdrop of high seroprevalence and asymptomatic transmission among households in Karachi, Pakistan.

## Introduction

An estimated 1.57 million cases of COVID-19 with 30,635 deaths have been reported in Pakistan until December 2022 [1]. The first three SARS-CoV-2 waves occurred from March to July 2020, October 2020 to January 2021, and March to May 2021 respectively. A fourth wave lasted from July to September 2021, was attributable to the delta variant and a fifth wave in December 2021 that lasted until February 2022 was due to the omicron variant [2], [3]. Majority of the cases in Pakistan (36%) have been reported from the southern province of Sindh and 21% of these occurred in its megacity, Karachi [1], [3].

High density urban dwellings with multigenerational housing increase the risk for transmission between all age groups within a household and serve as an ideal setting for studying the transmission dynamics of infectious diseases. Soon after the first confirmed COVID-19 case was recorded in Pakistan, a district-based surveillance system was set up for urgent diagnosis of cases and their isolation, tracing of contacts, coupled with the introduction of non-pharmacological interventions to contain the spread. This was based on the WHO strategy to test, trace and isolate [4]. The WHO at the same time published various protocols to investigate the dynamics of COVID-19 transmission on its website, which are intermittently updated [5]. We adopted one such protocol with the aim to study SARS-CoV-2 household transmission in the background of increasing seroprevalence due to the previous and ongoing COVID-19 wave.

## Methods

### Study design and setting

From October 2020 to January 2021, during the second wave of the pandemic, we conducted a case-ascertainment, prospective longitudinal study of confirmed COVID-19 cases and their household contacts in Pakistan to capture the transmission dynamics prior to introduction of vaccines in Pakistan. This study was carried out in District East of Karachi, which has a population of 2.9 million people residing in 509,647 households [6]. The field methods are derived from the published WHO protocols for the First Few X cases and contacts (FFX) [7].

### Ascertainment of index cases and household contacts

Cases were identified through the District Health Office database as those with a positive reverse-transcription polymerase chain reaction (RT-PCR) for SARS-CoV-2. Both hospitalized and non-hospitalized cases were eligible for enrollment while healthcare workers were excluded. Cases were approached within 72 h of the confirmation of their result.Those who consented were enrolled in the study along with all the household contacts. A household contact was defined as any person who has resided in the same household (or other closed setting) as a confirmed COVID-19 case. All the household contacts met the condition of proximity of less than 2 m with the confirmed case for a minimum of 15 min. We enrolled households with at least one contact (household size>=2).

### Household follow-up and data collection

Trained study staff collected epidemiological data on symptoms, medical history, and details of the exposure at the time of enrollment. Nasal swabs and blood samples were longitudinally collected from both the confirmed cases and their household contacts on Day 1 (enrollment) and days 7, 14, and 28.

Nasal swabs were collected from anterior nares using a flocked or spun polyester swab. The swabs were placed immediately into a sterile transport tube containing 2–3 ml of either viral transport medium (VTM). For serology, trained phlebotomists collected 5 ml of venous blood samples from each adult participant and 3 ml from infants. Both the nasal swabs and blood samples were maintained at a temperature of 2–8 °C and transported to the Aga Khan University Clinical lab.

All participants were additionally contacted daily via telephone to record new onset or resolution of symptoms. Contacts were additionally PCR-tested if they developed symptoms in between the scheduled visits as per the national guidelines.

### Laboratory analyses

Nasopharyngeal swab specimens were confirmed positive for SARS-CoV-2 by reverse transcription polymerase chain reaction (RT-PCR) using the SARS-CoV-2 Cobas 6800 Roche assay at the AKUH Clinical laboratories. Specimens with a cycle threshold (Ct) value less than or equal to 30 were defined as SARS-CoV-2 positive [8]. Serum samples were analyzed for the presence of anti-SARS-CoV-2 antibodies using Roche Anti SARS-CoV-2 antibody test which uses electrochemiluminescence immunoassay (ECLIA) for the in vitro qualitative detection of total SARS-CoV-2 antibodies targeting nucleocapsid (N) antigens [9].

### Sample size estimation

We estimated that a total of 95 cases and 570 contacts will be required for an overall secondary attack rate of 20% in the low-income/densely populated setting of district east with a margin of error of 5.0% and a confidence level of 95%.

### Statistical analysis

An index SARS-CoV-2 infected case was defined as having a positive reverse-transcription polymerase chain reaction (RT-PCR) test for SARS-CoV-2 virus. A secondary case was defined as any SARS-CoV-2 infection in a household contact not being the index case with a negative RT-PCR at day 1 and positive RT-PCR test during follow-up visits, regardless of symptom onset OR, SARS-CoV-2 negative serology at enrollment and positive serology at end of follow-up (seroconversion). A co-primary case was defined as any SARS-CoV-2 infection in a household member not being the index case, with positive RT-PCR test at enrollment (day 1) OR positive serology at enrollment and a history of recent (< 2 weeks before enrollment) respiratory symptoms. A susceptible contact was defined as any household contact with a negative RT-PCR test and a negative serology at enrollment (day 1). An uninfected contact was defined as any household contact who remained RT-PCR negative and serology negative from day 1 till day 28.

Secondary attack rate (SAR) was calculated as the proportion of total number of secondary infections among all susceptible contacts enrolled in the study.

$\mathrm{SAR}=\frac{\mathrm{total number of secondary cases identified}}{\mathrm{total number of susceptible contacts}}X100$

To control the effect of co-primary cases on household transmission, we performed a sensitivity analysis, by calculating SAR in two ways: first, by excluding all households with any co-primary case. In the second approach, we assumed all primary (including co-primary) cases contributed to the infection transmission independently, and calculated SARs for each of the households accounting for the number of primary and co-primary cases. We reported the mean SAR accounting for all primary cases (including co-primary cases) across all the households. The effective household case cluster size was estimated as the average number of secondary cases per infectious case in a household setting with no co-primary cases [10].

$\mathrm{Effective household case cluster size}=\frac{\mathrm{total number of secondary cases across all households}}{\mathrm{total number of primary cases}}$

We used a binomial logistic regression model to determine the effect of various household and non-household related epidemiological factors on the odds of SARS-CoV-2 transmission among household members. All factors which had a p-value of < 0.25 at univariate level were included in the multivariate analysis. A backward elimination method was used to derive a parsimonious model where all factors had a p-value less than 0.05. All analysis was performed in the statistical software package STATA version 15.0.

## Ethics

Data collection was initiated after obtaining ethical approval from the Ethical Review Committee of the Aga Khan University, Karachi, Pakistan (ERC no. 2020–5432-14139). Written informed consent and assent (where applicable) were taken from individual participants.

## Results

### Characteristics of index cases and contacts

**Fig. 1:**
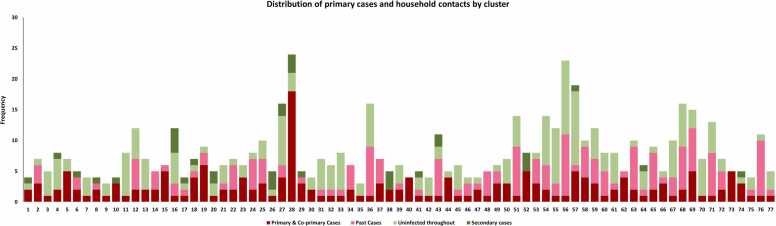
Household-wise distribution of primary cases and household contacts (co-primary cases, non-susceptible contacts, secondary cases, and uninfected susceptible contacts).

As per the health office records, 6195 SARS-CoV-2 confirmed cases were reported between October 2020 and January 2021 in the district east of Karachi. Of these, 695 cases were approached within 72 h of the confirmation of their result and 77 (11.0%) provided household level consent to participate in the study. In these 77 households, there were 683 contacts identified, out of which, 543 consented to be part of the study. The distribution of these contacts across the household is shown in Fig. 1.

**Fig. 2:**
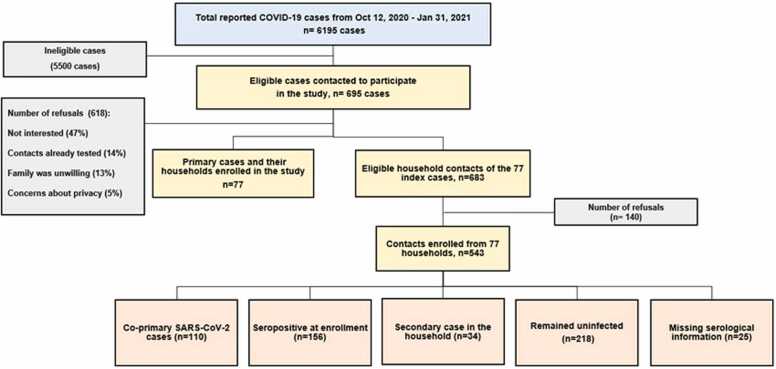
Flow of participants in the study.

**Fig. 3:**
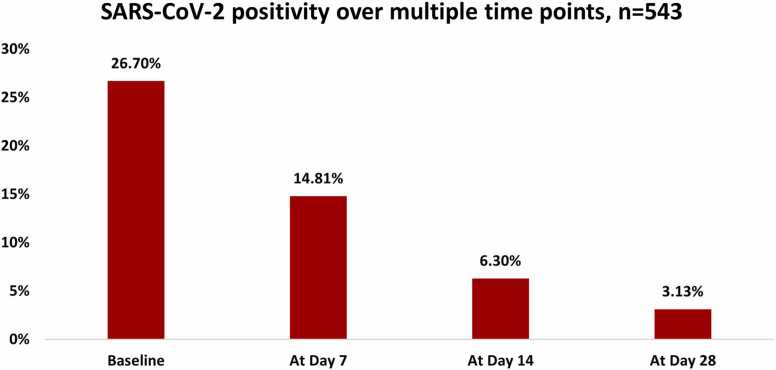
SARS-CoV-2 positivity at various time points (at enrollment, days 7, 14 and 28).

Table 1 describes the characteristics of the index cases and their household contacts. The median household cluster size was 6.0 (IQR 5.0–8.0). The mean ( ± SD) age of index cases was 41.4 ± 15.9 years and 12.9% were symptomatic at the time of enrollment and none were hospitalized. Among the index cases, almost half 40 (51.9%) had participated in a festival or mass gathering before the onset of their symptoms. In the two weeks prior to diagnosis, 36 cases (46.7%) had encountered a person with a similar illness, while 15.6% were exposed within their own homes and 14.3% were exposed at their workplace and 88.3% of the primary cases always wore masks outside of their homes. Contacts had a mean ( ± SD) age of 31.0 ± 19.8 years and 10.4% experienced symptoms. Among the 543 contacts, an additional 110 (20.8%) had a positive RT-PCR test at enrollment (co-primary cases) and 156 (32.9%) had evidence of a previous infection through detectable SARS-CoV-2 IgG antibodies. For 25 contacts, the serological information was missing. Over the course of study follow up, there were 34 secondary cases from 252 susceptible contacts (secondary attack rate 14.0%, 95% CI 9.0–18.0). Sensitivity analysis showed that the SAR was 10.7% (95% CI 6.6–14.8) when households with co-primary cases were excluded, and the mean SAR accounting for all primary and co-primary cases was 22.7% (95% CI 18.1–27.3). Of the 77 households, transmission took place in 20 (25.9%). The flow of participants in the study is described in Fig. 2. At enrollment, 26.7% (n-161) of the samples tested positive for COVID-19 and decreased to 3.13% (n = 18) by day 28 (Fig. 3).

**Table 1:** Sociodemographic characteristics of primary cases and their household contacts (including co-primary cases, non-susceptible contacts, secondary cases, and uninfected susceptible contacts).

**Characteristics**	**Primary cases**	**Contacts**	**Total**
	**N = 77**	**N = 543**	**N = 620**
**Age, years, (mean± SD)**	41.38 ± 15.90	31.09 ± 19.87	32.37 ± 19.70
0-10 Years	1 ( 1.30%)	92 (16.94%)	93 (15.00%)
11-20 Years	4 ( 5.19%)	99 (18.23%)	103 (16.61%)
21-30 Years	15 (19.48%)	100 (18.42%)	115 (18.55%)
31-40 Years	21 (27.27%)	98 (18.05%)	119 (19.19%)
41-50 Years	17 (22.08%)	46 ( 8.47%)	63 (10.16%)
51-60 Years	8 (10.39%)	46 ( 8.47%)	54 ( 8.71%)
> 60 Years	11 (14.29%)	62 (11.42%)	73 (11.77%)
**Gender**			
Male	45 (58.44%)	270 (49.72%)	315 (50.81%)
Female	32 (41.56%)	273 (50.28%)	305 (49.19%)
**Employment status**			
Employed	44 (57.14%)	181 (33.33%)	225 (36.29%)
Unemployed	33 (42.86%)	362 (66.67%)	395 (63.71%)
**Household information, n = 77**			
Household size (number of people who usually live in the house), median (IQR)	6.0 (5.0-8.0)	-	6.0 (5.0-8.0)
Total number of rooms in house (including kitchen but not including bathrooms), median (IQR)	4.0 (3.0-5.0)	-	4.0 (3.0-5.0)
Number of bedrooms, median (IQR)	3.0 (3.0-4.0)	-	3.0 (3.0-4.0)
**Symptoms**			
Fever	15 (19.48%)	85 (15.65%)	100 (16.13%)
Sore throat	27 (35.06%)	97 (17.86%)	124 (20.00%)
Congestion or runny nose	19 (24.68%)	119 (21.92%)	138 (22.26%)
Cough	33 (42.86%)	126 (23.20%)	159 (25.65%)
Shortness of breath	18 (23.38%)	34 ( 6.26%)	52 ( 8.39%)
Nausea or vomiting	5 ( 6.49%)	13 ( 2.39%)	18 ( 2.90%)
Diarrhea	5 ( 6.49%)	9 ( 1.66%)	14 ( 2.26%)
Headache	21 (27.27%)	80 (14.73%)	101 (16.29%)
Muscle/Body ache	29 (37.66%)	72 (13.26%)	101 (16.29%)
Loss of smell	29 (37.66%)	72 (13.26%)	101 (16.29%)
Loss of taste	19 (24.68%)	41 ( 7.55%)	60 ( 9.68%)
Fatigue	27 (35.06%)	69 (12.71%)	96 (15.48%)
Other symptoms	12 (15.58%)	16 ( 2.95%)	28 ( 4.52%)
Pre-existing comorbidities*	18 (23.38%)	62 (11.42%)	80 (12.90%)
**Human exposures in the days before symptom onset (in the past 14 days)**			
Domestic Travel History	6 ( 7.79%)	23 ( 4.24%)	29 ( 4.68%)
			
International Travel History	0 ( 0.00%)	2 ( 0.37%)	2 ( 0.32%)
In the past 14 days did you attend festival or mass gathering? n = 77	40 (51.95%)	-	40 (51.95%)
			
In the past 14 days were you exposed to a person with similar illness? n = 77	36 (46.75%)	-	36 (46.75%)
			
Location of exposure in the past 14 days, n = 77			
Home	12 (15.58%)	-	12 (15.58%)
Workplace	11 (14.29%)	-	11 (14.29%)
Tour group	13 (16.88%)	-	13 (16.88%)
**Use of mask**			
Do you wear a face mask every time you go out?			
Never	0 ( 0.00%)	22 ( 4.05%)	22 ( 3.55%)
Sometimes	9 (11.69%)	62 (11.42%)	71 (11.45%)
Always	68 (88.31%)	459 (84.53%)	527 (85.00%)

Employed: government job, private job, daily wages worker and self-employed

Un-employed: students and housewives

* Co-morbid conditions include cancer, Diabetes, HIV or other immune deficiency, heart disease, asthma, chronic lungs disease, chronic kidney disease, chronic anemia disease, chronic neurological disease, organ or bone marrow recipient and other pre-existing conditions

**Fig. 4:**
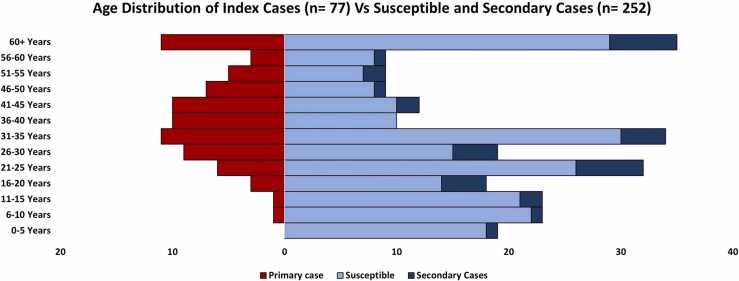
Age distribution of index cases (n = 77) vs susceptible and secondary cases (n = 252).

Table 2 gives the comparison of susceptible contacts who went on to develop a secondary infection versus those who remained uninfected. Secondary cases had a mean ( ± SD) age of 35.3 ± 19.7 years and 20 (58.4%) were adults aged ≥ 18 years, 6 (17.6%) were above 60 years of age and 8 (23.4%) were less than 20 years (Fig. 4). Most secondary cases were female (55%), unemployed (67.5%) and 61% had 5–10 members in their household. Of the 34 secondary cases detected in 20 households post-enrollment, 16 (47%) occurred before the second follow-up at 14 days. Secondary infections occurred throughout the study period, but most were clustered in October (n = 14, 41%). During their infection, 55.9% of the participants reported symptoms. The most reported symptoms among secondary cases were fever (n = 12, 35.3%), sore throat (n = 12, 35.3%), nasal congestion/runny nose (n = 13, 38.2%), cough (n = 12, 35.3%), fatigue (n = 13, 38.2%) and loss of smell (n = 12, 35.3%) (Table 2). We did not find any significant differences with regards to age, gender, and previous comorbidities between secondary cases and household contacts who did not get infected.

**Table 2:** Sociodemographic and household interactions of Non-Cases (uninfected contacts) and Secondary Cases. The table presents Odds Ratios (OR) with 95% Confidence Intervals (CI) for sociodemographic variables associated with secondary transmission.

**Characteristics**	**Non-cases**	**Secondary cases**	**OR (CI)**
	**N = 218**	**N = 34**	
**Age, years, (mean± SD)**	30.62 ± 19.92	35.30 ± 19.67	1.01(0.99,1.03)
0-10 Years	40 (18.35%)	2 ( 5.88%)	Ref
11-20 Years	35 (16.06%)	6 (17.65%)	3.43(0.65,18.09)
21-30 Years	41 (18.81%)	10 (29.41%)	4.88(1.01,23.67)
31-40 Years	40 (18.35%)	4 (11.76%)	2.0(0.35,11.54)
41-50 Years	18 ( 8.26%)	3 ( 8.82%)	3.33(0.51,21.71)
51-60 Years	15 ( 6.88%)	3 ( 8.82%)	4.0(0.61,26.35)
> 60 Years	29 (13.30%)	6 (17.65%)	4.14(0.78,21.99)
**Gender**			
Male	109 (50.00%)	15 (44.12%)	Ref
Female	109 (50.00%)	19 (55.88%)	1.27(0.61,2.62)
**Employment status**			
Employed	83 (38.07%)	11 (32.35%)	0.78(0.36,1.68)
Unemployed	135 (61.93%)	23 (67.65%)	Ref
**Symptoms**			
Fever	23 (10.55%)	12 (35.29%)	4.62(2.03,10.56)
Sore throat	30 (13.76%)	12 (35.29%)	3.42(1.53,7.62)
Congestion or runny nose	43 (19.72%)	13 (38.24%)	2.52(1.17,5.43)
Cough	39 (17.89%)	12 (35.29%)	2.50(1.14,5.48)
Nausea or vomiting^@^	3 ( 1.38%)	3 ( 8.82%)	6.94(1.34,35.9)
Headache	19 ( 8.72%)	11 (32.35%)	5.01(2.12,11.82)
Muscle/Body ache	17 ( 7.80%)	12 (35.29%)	6.45(2.73,15.24)
Loss of smell	17 ( 7.80%)	12 (35.29%)	6.45(2.73,15.24)
Loss of taste	5 ( 2.29%)	8 (23.53%)	13.11(3.99,43.05)
Fatigue^@^	14 ( 6.42%)	13 (38.24%)	9.02(3.75,21.71)
Pre-existing comorbidities*	22 (10.09%)	5 (14.71%)	1.54(0.54,4.37)
**Household size**^**%**^			
< 5 members	44 (20.18%)	9 (26.47%)	Ref
5-10 members	141 (64.68%)	21 (61.76%)	0.73(0.31,1.71)
> 10 members	33 (15.14%)	4 (11.76%)	0.59(0.17,2.09)
**Domestic Travel History**	9 ( 4.13%)	2 ( 5.88%)	1.45(0.3,7.02)
**International Travel History**	1 ( 0.46%)	1 ( 2.94%)	6.58(0.4107.69)
**Contact typically shares a room with the primary case, n = 251**	110 (50.69%)	17 (50.00%)	0.97(0.47,2.00)
**Contact Duration with primary case, Median (IQR)**	1.0 (0.0-5.0)	0.5 (0.0-3.0)	0.94(0.84,1.04)
**Contact took care of primary case at home during illness**	68 (31.19%)	14 (41.18%)	1.54(0.74,3.24)
**Contact hugged primary case during illness, n = 250**	66 (30.41%)	6 (18.18%)	0.51(0.2,1.29)
**Contact shook hands with primary case during illness n = 247**^@^	132 (60.83%)	13 (43.33%)	0.49(0.23,1.07)
**Contact shared a meal with primary case during illness**	122 (55.96%)	18 (52.94%)	0.89(0.43,1.83)
**Contact ate with hands from the same plate as primary case during illness**	39 (17.89%)	5 (14.71%)	0.79(0.29,2.17)
**Contact shared a drinking cup/glass with primary case during illness n = 250**	54 (25.00%)	9 (26.47%)	1.08(0.48,2.46)
**Contact shared utensils with primary case during illness**	72 (33.03%)	9 (26.47%)	0.73(0.32,1.65)
**Contact slept in the same room as primary case during illness**	37 (16.97%)	7 (20.59%)	1.27(0.51,3.13)
**Contact shared a toilet with case during illness, n = 249**	60 (27.65%)	10 (31.25%)	1.19(0.53,2.66)
**Contact wears face mask every time outside the house**			
Never	10 ( 4.59%)	0 ( 0.00%)	-
Sometimes	29 (13.30%)	2 ( 5.88%)	0.39(0.09,1.70)
Always	179 (82.11%)	32 (94.12%)	Ref

@Final adjusted model was significant for Nausea/vomiting aOR: 7.89 95% CI 1.37,45.53, Fatigue aOR:9.31, 95% CI 3.81,22.78 and did the contact shake hands with the case during the time he/she was ill ? aOR: 0.41 95% CI 0.17,0.97

%Household size refers to the number of people in the household of each contact.

*Co-morbid conditions include cancer, Diabetes, HIV or other immune deficiency, heart disease, asthma, chronic lungs disease, chronic kidney disease, chronic anemia disease, chronic neurological disease, organ or bone marrow recipient and other pre-existing conditions.

Employed: government job, private job, daily wages worker and self-employed and un-employed: retired persons, students, and housewives

Among the household contacts, 17 (50%) secondary cases and 110 (50%) non-cases shared a room with the primary case, 14 (41.1%) secondary cases and 68 (31.1%) non-cases provided care to the primary case during their illness, 7 (20.5%) of secondary cases slept in the same room as the primary case during their illness, in contrast to 37 (16.9%) of non-cases. Furthermore, 30 (31.2%) of secondary cases shared a toilet with the primary case during their illness, whereas 60 (27.6%) of non-cases did the same. A majority, 32 (94.1%) of secondary cases consistently wore masks outside their homes, compared to 179 (82.1%) of the non-cases. *Effective household case cluster size*.

The effective household case cluster size was 0.46 (95%CI 0.33,0.56). For cases recorded before the peak of second wave (8th December 2020) in Pakistan, it was 0.50 (95%CI 0.3,0.7) with a corresponding pre-peak SAR of 21% (95% CI 13.0,29.0). We estimated the effective household case cluster size after 8th December to be 0.40 (95% CI 0.15,0.65) with a SAR of 9% (95% CI 5.0,14.0).

### Risk factors for transmission of SARS-CoV-2

Table 2 gives the odds of secondary transmission in the household contacts by a range of explanatory variables. In the unadjusted model, secondary cases were more likely to have provided care to the case during the time he/she was ill at home (OR, 1.54; 95% CI, 0.74–3.24) and to have slept in the same room as the primary case (OR, 1.27; 95% CI, 0.51–3.13) than non-cases. However, these associations were not statistically significant. Secondary cases were more likely to have symptoms of nausea and vomiting (aOR, 7.9; 95% CI, 1.4–45.5) and fatigue (aOR, 9.3; 95% CI, 3.8–22.7) than uninfected contacts.

Table 3 describes the various predictors for non-household transmission of SARS-CoV-2 infection. Cancelling or delaying overseas travel was protective against secondary transmission (OR, 0.37; 95% CI, 0.14–0.97).

**Table 3:** Predictors for non-household transmission of SARS-CoV-2 infection among secondary cases and uninfected contacts.

**Social distancing activities undertaken**	**N = 252**		**OR (CI)** **(N = 98)**	**P-value**
	**Non-cases**	**Secondary cases**		
	**N = 218**	**N = 34**		
**Deliberately cancelled or postponed a social event, n = 148**				
Yes	81 (65.32%)	19 (79.17%)	1.82(0.73,4.59)	0.202
No	43 (34.68%)	5 (20.83%)	Ref	
**Cancelled or delayed travelling overseas, n = 107**				
Yes	52 (57.78%)	5 (29.41%)	0.37(0.14,0.97)	0.042
No	38 (42.22%)	12 (70.59%)	Ref	
**Reduced the use of public transport, n** = **144**				
Yes	100 (81.97%)	19 (86.36%)	1.33(0.43,4.16)	0.623
No	22 (18.03%)	3 (13.64%)	Ref	
**Increased the amount of household products purchased, n** = **161**				
Yes	72 (53.33%)	19 (73.08%)	2.09(0.93,4.69)	0.074
No	63 (46.67%)	7 (26.92%)	Ref	
**Parents kept one or more of their children out of school or pre-school, n = 108**				
Yes	41 (45.05%)	8 (47.06%)	1.07(0.45,2.56)	0.879
No	50 (54.95%)	9 (52.94%)	Ref	
**Kept children home during school closure, n = 153**				
Never	10 ( 7.87%)	3 (11.54%)	Ref	
Always	99 (77.95%)	18 (69.23%)	0.67(0.23,1.96)	0.462
Sometimes	18 (14.17%)	5 (19.23%)	0.94(0.27,3.32)	0.926
**Kept away from crowded places generally, n = 162**				
Yes	113 (83.09%)	21 (80.77%)	0.88(0.36,2.13)	0.773
No	23 (16.91%)	5 (19.23%)	Ref	
**Increased the time I spent cleaning or disinfect things I might touch, such as d, n=161**				
Yes	93 (68.89%)	20 (76.92%)	1.42(0.61,3.31)	0.421
No	42 (31.11%)	6 (23.08%)	Ref	
**Washed my hands with soap and water more often than usual, n = 168**				
Yes	118 (83.10%)	23 (88.46%)	1.47(0.47,4.55)	0.506
No	24 (16.90%)	3 (11.54%)	Ref	
**Used alcoholic hand gel more than usual, or hand sanitizer, n = 171**				
Yes	105 (71.92%)	20 (80.00%)	1.47(0.59,3.69)	0.41
No	41 (28.08%)	5 (20.00%)	Ref	
**Increased my use of vitamins or oral supplements, n = 163**				
Yes	76 (55.47%)	17 (65.38%)	1.42(0.67,3)	0.355
No	61 (44.53%)	9 (34.62%)	Ref	
**Worked from home, n = 146**				
Never	26 (20.97%)	3 (13.64%)	Ref	
Always	73 (58.87%)	15 (68.18%)	1.65(0.51,5.29)	0.401
Sometimes	25 (20.16%)	4 (18.18%)	1.33(0.33,5.44)	0.688
**Stayed in quarantine at home (until test results were available), n = 173**				
Never	13 ( 8.97%)	2 ( 7.14%)	Ref	
Always	116 (80.00%)	23 (82.14%)	1.24(0.32,4.75)	0.753
Sometimes	16 (11.03%)	3 (10.71%)	1.18(0.23,6.2)	0.841
**Avoided going to a large event like a sports match, n = 175**				
Never	14 ( 9.40%)	0 ( 0.00%)	Ref	
Always	120 (80.54%)	23 (88.46%)	0.97(0.32,2.9)	0.949
Sometimes	15 (10.07%)	3 (11.54%)	-	

## Discussion

We describe the transmission dynamics of SARS-CoV-2 in 77 households during the second COVID-19 wave in Pakistan. We report a secondary attack rate (SAR) of 14.0% within households and an effective household case cluster size of 0.46, indicating lower transmissibility among the observed contacts. In addition, the risk for secondary transmission was higher in contacts who reported symptoms of nausea, vomiting and fatigue whereas the risk was lower in contacts who cancelled or delayed overseas travel.

Our estimated secondary attack rate was comparable with the typical SAR of 16.6% (95% CI 14.0%−19.3%) as reported in a meta-analysis of 54 household transmission studies [11]. When households with co-primary cases were excluded, the SAR was 10.7%, while it increased to 22.7% when co-primary cases and their households were included. These results suggest a likely range for the true SAR estimates lying between these values. Previous household SAR during the first few months of the pandemic has ranged from 5% to 30% in China, Taiwan, India, and Bangladesh [12], [13], [14], [15]. The variation in SAR across studies can be attributable to differences in their study designs, household size and composition, prevention measures adopted by the families and contact tracing.

In our study, the enrollment of cases and their household contacts commenced in early October 2020, before introduction of vaccine and in the backdrop of easing of government restrictions and increasing non-compliance towards social distancing and masking. Consequently, the positivity rate increased till 8%− 11% and a sudden increase in the number of active cases and hospital admissions were recorded [1]. Despite this we observed that secondary transmission from a primary case occurred in one-quarter of the households and one in seven susceptible contacts (i.e., secondary attack rate 14%). These low transmission levels might be partly due to the impact of containment strategies, non-pharmaceutical interventions, and existing immunity due to previous symptomatic or asymptomatic transmission. At the time of enrollment, (156/543) 32.9% of the household contacts were seropositive for SARS-CoV-2. We previously reported a seroprevalence of 24% during the same period which increased up to 53.9% by early February 2021 [16]. This then resulted in depletion of the susceptible population thus reducing transmission until the emergence of new variants.

Our data shows that index cases were more likely to be adult males which is consistent with the higher incidence of the disease reported among males [17]. Female contacts were more susceptible to acquiring the infection secondarily from a male in the household, similar to a review by Zachary et al. [18]. The landmark HOSTED study from UK also noted higher rates of secondary transmission of COVID-19 among female contacts. This predisposition of female contacts to infection may be because of the traditional role of women as the caregiver of the family [19]. We enrolled 65 contacts aged less than 15 years of which 4 children developed secondary infection. Previous studies have shown that children are mostly asymptomatic or exhibit mild symptoms with faster recovery and could thus be less likely to be tested as compared to older individuals [17], [19]. This could have contributed to the lower proportion of the infection in this age group.

The overall proportion of infected contacts who were asymptomatic (44.1%) was higher than a previous estimate, 17% (95% CI 13%−20%) of outbreaks in closed or residential settings [20]. Our study showed that symptoms of nausea/vomiting and fatigue in infected contacts were associated with secondary transmission consistent with prior literature [21].

This is the first community-based study from Pakistan that prospectively ascertained SARS-CoV-2 transmission among a susceptible population. We adopted a standardized WHO protocol and performed entire household testing using both RT-PCR and serology tests during four follow-up household visits over a month. We were able to confirm current illness irrespective of symptoms. Secondly, a meticulous record of symptoms was maintained through daily phone calls for each participant during the entire follow-up period through which were able to ascertain temporality i.e., onset of infection following exposure.

Our study had some limitations. Of the 695 index cases approached, only 11% consented to participate in the study, thus, there was a limited number of family clusters studied. At the time of inclusion, (110/543) 20.8% of the household contacts had tested positive for the virus (RT-PCR) and a majority of these were asymptomatic. These results may imply that they had been infected concurrently or before the index cases. There may be a possibility that contacts acquired the infection from the community due to non-random mixing between households and different risk groups. We have reported various social distancing activities undertaken by the contacts. However, we could not perform a complete case analysis for the non-household transmission variables due to missing data. There may be survivor bias as only cases who had mild to moderate disease were enrolled in the study. None of the cases in our study were hospitalized before or during the study period. It is possible that milder cases of the disease may not have a sufficient viral load to spread the infection efficiently and that could have resulted in the lower SAR estimate.

## Conclusion

We observed low SARS-CoV-2 transmission in the backdrop of high seroprevalence among households in Karachi, Pakistan in the early months of the pandemic. High proportion of asymptomatic cases and low diagnostic testing is likely to be attributable to the gap between seroprevalence and reported cases. These findings highlight the importance of asymptomatic infection and transmission. Our observed transmission dynamics can be coupled with SARS-CoV-2 evolution patterns obtained through large-scale pathogen whole genome sequencing (WGS) and phylogenetic analysis to design strategies for diagnosis, therapeutic intervention, and the development of next generation vaccines.

## Funding

This work was supported by the 10.13039/100000865Bill & Melinda Gates Foundation, Seattle, WA (grant number grant ID #INV-021777). The authors declare that no funds, grants, or other support were received during the preparation of this manuscript.

## Declaration of Competing Interest

The authors have no relevant financial or non-financial interests to disclose.
