# Supplementary material for: Secondary attack rates and determinants of Severe Acute Respiratory Syndrome Coronavirus 2 (SARS-CoV-2) household transmission in Pakistan: A case-ascertained prospective, longitudinal study
Source: J Infect Public Health. 2024 May;17(5):889–96. doi: 10.1016/j.jiph.2024.03.024 (PMC11009119; doi:10.1016/j.jiph.2024.03.024)
Supplement: Supplementary file 1 — Supplementary material [file mmc1.docx]

***Form 1A: Case initial reporting from – for confirmed cases (Day 1)***

**Unique Primary Case ID: __________________**

**Household number: ______________________**

| **1. Data collector information** |  |
| --- | --- |
| Name/code of data collector |  |
| Data collector telephone number |  |
| Visit date (dd/mm/yyyy) | __/__/__ |
| **2. Interview respondent information**  (if the person providing the information is not the primary case; skip if case is the respondent) | |
| First name |  |
| Family name |  |
| Gender | □ Male □ Female |
| Date of birth (dd/mm/yyyy) | ___/___/___ |
| Age | Years ____ ____ Months ____ ___ |
| Relationship to primary case |  |
| Respondent address/GIS coordinates |  |
| Telephone (mobile) number |  |
| **3. Primary case identifier information** |  |
| First Name |  |
| Family Name |  |
| Gender | □ Male □ Female |
| Date of birth (dd/mm/yyyy) | ___/___/___ |
| Telephone (mobile) number |  |
| Age (years, months) | ___ years ___ months |
| Address/GIS coordinates |  |
| National identity number (CNIC) (if applicable) |  |
| Country of residence |  |
| Nationality |  |
| Ethnicity |  |
| Case Occupation | 11= Government job  12= Private job  13= Daily wages worker  14= Self employed  15= Farmer  16= Does not work  17= Other  99=Not applicable |

| **4. Household information** |  |
| --- | --- |
| Household size  (number of people who usually live in the house) |  |
| Total number of rooms in house  (including kitchen but not including bathrooms) |  |
| Number of bedrooms |  |
| List of household members  (eldest to youngest) with age and gender  (multiple response) | 1. Name______________   Age: ______________  Gender: _____________   1. Name______________   Age: ______________  Gender: _____________ |

| **5a. Primary case symptoms (from onset of symptoms)** |  |
| --- | --- |
| Date of first symptom onset (dd/mm/yyyy) | ___/___/___ |
| Fever (≥38 °C) or history of fever | □ Yes □ No □ Unknown |
|  | If Yes, specify maximum temperature: °C |
| Date of first health facility visit (including traditional care) | ___/___/___ |
|  | □ Not applicable (na) □ Unknown |
| Total health facilities visited to date | Specify number:_________ □ na □ Unknown |
|  |  |
| **5b. Respiratory symptoms** |  |
| Sore throat | □ Yes □ No □ Unknown  If Yes, date of onset (dd/mm/yyyy): ___/___/___ |
| Runny nose | □ Yes □ No □ Unknown  If yes, (dd/mm/yyyy): ___/___/___ |
| Cough | □ Yes □ No □ Unknown  If Yes, date of onset (dd/mm/yyyy): ___/___/___ |
| Shortness of breath | □ Yes □ No □ Unknown  If Yes, date of onset (dd/mm/yyyy): ___/___/___ |
| **5c. Other symptoms** |  |
| Chills | □ Yes □ No □ Unknown |
| Vomiting | □ Yes □ No □ Unknown |
| Nausea | □ Yes □ No □ Unknown |
| Diarrhoea | □ Yes □ No □ Unknown |
| Headache | □ Yes □ No □ Unknown |
| Rash | □ Yes □ No □ Unknown |
| Conjunctivitis | □ Yes □ No □ Unknown |
| Muscle aches | □ Yes □ No □ Unknown |
| Joint ache | □ Yes □ No □ Unknown |
| Loss of appetite | □ Yes □ No □ Unknown |
| Loss of smell (anosmia) | □ Yes □ No □ Unknown |
| Loss of taste | □ Yes □ No □ Unknown |
| Nose bleed | □ Yes □ No □ Unknown |
| Fatigue | □ Yes □ No □ Unknown |
| Seizures | □ Yes □ No □ Unknown |
| Altered consciousness | □ Yes □ No □ Unknown |
| Other symptoms | □ Yes □ No □ Unknown |
|  | If Yes, specify: |

| **6. Primary case pre-existing condition(s)** |  |
| --- | --- |
| Pregnancy | □ Yes □ No □ Unknown |
|  | If Yes, specify trimester: |
|  | □ First □ Second □ Third □ Unknown |
| Obesity | □ Yes □ No □ Unknown |
| Cancer | □ Yes □ No □ Unknown |
| Diabetes | □ Yes □ No □ Unknown |
| HIV/other immune deficiency | □ Yes □ No □ Unknown |
| Heart disease | □ Yes □ No □ Unknown |
| Asthma (requiring medication) | □ Yes □ No □ Unknown |
| Chronic lung disease (non-asthma) | □ Yes □ No □ Unknown |
| Chronic liver disease | □ Yes □ No □ Unknown |
| Chronic haematological disorder | □ Yes □ No □ Unknown |
| Chronic kidney disease | □ Yes □ No □ Unknown |
| Chronic neurological impairment/disease | □ Yes □ No □ Unknown |
| Organ or bone marrow recipient | □ Yes □ No □ Unknown |
| Other pre-existing condition (s) | □ Yes □ No □ Unknown |
|  | If Yes, specify: |

| **7. Clinical course: Complications** |  |
| --- | --- |
| Hospitalization required? | □ Yes □ No □ Unknown |
|  | If yes, name of hospital  Date of admission: __/__/___ |
| ICU (intensive care unit) admission | □ Yes □ No □ Unknown |
| If yes, Date of ICU admission (dd/mm/yyyy) | ___/___/___ □ Unknown |
| Date of discharge from ICU (dd/mm/yyyy) | ___/___/___ □ Unknown |
| Mechanical ventilation | □ Yes □ No □ Unknown |
| Dates of mechanical ventilation (dd/mm/yyyy) | Start:___/___/___ □ Unknown |
|  | Stop: ___/___/___ □ Unknown |
| Acute respiratory distress syndrome (ARDS) | □ Yes □ No □ Unknown |
|  | If Yes, date started (dd/mm/yyyy)___/___/___ |
| Acute renal failure | □ Yes □ No □ Unknown |
|  | If Yes, date started (dd/mm/yyyy)___/___/___ |
| Cardiac failure | □ Yes □ No □ Unknown |
|  | If Yes, date started (dd/mm/yyyy)___/___/___ |
| Consumptive coagulopathy | □ Yes □ No □ Unknown |
|  | If Yes, date started (dd/mm/yyyy)___/___/___ |
| Pneumonia by chest X-ray | □ Yes □ No □ Unknown |
|  | If Yes, date started (dd/mm/yyyy) ___/___/___ |
| Other complications | □ Yes □ No □ Unknown |
|  | If Yes, specify: |
| Hypotension requiring vasopressors | □ Yes □ No □ Unknown |
| Extracorporeal membrane oxygenation (EMO) required | □ Yes □ No □ Unknown |
| Outcome | Currently isolating at home  Currently admitted in hospital  Recovered completely at home  Recovered completely at hospital  Recovered and discharged from hospital  Discharged against medical advice  Death |
| Date of discharge from hospital (if applicable)  (dd/mm/yyyy) | ___/___/___ |
| Outcome current as of date (dd/mm/yyyy) | ___/___/___ |
|  | □ Unknown □ na |

| **8. Health-care interactions** |  |
| --- | --- |
| Contact with emergency number/ hotline | □ Yes □ No □ Unknown |
| Date of emergency contact (dd/mm/yyyy) | ___/___/___ □ Unknown |
| Visit to primary healthcare (PHC; GP, etc.) (repeat for as many visits with dates as required) | 1. □ Yes □ No □ Unknown   If yes: ___/___/___   1. □ Yes □ No □ Unknown   If yes: ___/___/___   1. □ Yes □ No □ Unknown   If yes: ___/___/___ |
| Visited emergency department (A&E) (repeat for as many visits as required) | 1. □ Yes □ No □ Unknown   If yes: ___/___/___   1. □ Yes □ No □ Unknown   If yes: ___/___/___   1. □ Yes □ No □ Unknown   If yes: ___/___/___ |
| Hospitalization (repeat for as many admissions as required) | 1. □ Yes □ No □ Unknown   If yes, Specify:  Name of hospital:__________  Admission date:_ __/___/___  Discharge date: __/___/___   1. □ Yes □ No □ Unknown   If yes, Specify:  Name of hospital: __________  Admission date: _ __/___/___  Discharge date: __/___/___ |

| **9. Human exposures in the days before symptom onset (in the past 14 days)** | |
| --- | --- |
| Have you travelled **domestically** within the last 14 days? | □ Yes □ No □ Unknown |
|  | If yes Date of travel  ___/___/___ to ___/___/___ |
|  | Cities visited: (Multiple response possible)  ____________________________________ |
| Have you travelled **internationally** within the last 14 days? | □ Yes □ No □ Unknown |
|  | If Yes, please specify place and dates of travel (dd/mm/yyyy): (multiple response possible)   1. Name of city and country visited:   ___/___/___ to ___/___/___   1. Name of city and country visited:   ___/___/___ to ___/___/___   1. Name of city and country visited:   ___/___/___ to ___/___/___ |
| In the past 14 days, have you had contact with anyone with suspected or confirmed COVID-19 infection? | □ Yes □ No □ Unknown |
|  | If Yes, dates of last contact (dd/mm/yyyy): ___/___/___ |
| In the past 14 days, did you attend attended festival or mass gathering? | □ Yes □ No □ Unknown |
| In the past 14 days, were you exposed to person with similar illness ? | □ Yes □ No □ Unknown |
| Location of exposure in the past 14 days | □ Home □ Hospital □ Workplace  □ Tour group □ School □ Unknown  □ Other, specify: |

| **10. Use of Mask** |  |
| --- | --- |
| Do you wear a face mask every time you go out? | 󠇫 Yes 󠇫always 󠇫 Wear it Sometimes 󠇫 󠇫 Never worn a mask |
|  |  |
| Type of mask you usually wear | 󠇫 S**urgical mask**  How often do you use surgical masks?   - Single time - More than once   󠇫 **Cloth mask**  How often do you wash masks?   - ____ times/day - ____ times/week - ____ times/month   󠇫 **Respirator** 󠇫 **Other**, specify ___________________ |
|  |  |
| **11. Lab sample collection** |  |
| Blood sample has been collected | Yes □ No □ |
|  |  |
| Nasal swab sample has been collected | Yes □ No □ |
|  | If yes to any of the two above,  Date of collection of samples: DD/MM/YY  Laboratory identification number: ______________ |
| **12. Status of form completion** |  |
| Form completed | □ Yes □ No □ partially filled |
|  | Specify reason only if No or partially filled: |
|  | □ Missed |
|  | □ Not attempted |
|  | □ Not performed |
|  | □ Refusal |
|  | □ Other, specify: |

***Form 1B: Contact initial reporting form – for household contacts of confirmed cases (Day 1)***

**Unique Primary Case ID/Household number**

**Household Contact ID Number (C…):**

| **1. Data collector information** |  |
| --- | --- |
| Name/code of data collector |  |
| Data collector telephone number |  |
| Form completion date (dd/mm/yyyy) |  |
| **2. Interview respondent information (if the person providing the information is not the primary case)** |  |
| First name |  |
| Family name |  |
| Gender | □ Male □ Female |
| Date of birth (dd/mm/yyyy) | ___/___/___ |
| Relationship to primary case |  |
| Respondent address/GIS coordinates |  |
| Telephone (mobile) number |  |
| **3. Contact identifier information** |  |
| First name |  |
| Family name |  |
| Gender | □ Male □ Female |
| Date of birth (dd/mm/yyyy) | ___/___/___ |
| Relationship to confirmed case |  |
| Telephone (mobile) number |  |
| Age (years, months) | ___ years ___ months |
| Address/GIS coordinates | ---------------------------------------------------- |
| National identity number (CNIC)(if applicable) |  |
| Country of residence |  |
| Nationality |  |
| Ethnicity (optional) |  |

| **4a.** **General Exposure Information** |  |
| --- | --- |
| Have you travelled domestically within the last 14 days? | □ Yes □ No □ Unknown |
|  | If Yes, please specify:  Name of city: ___________  Dates of travel (dd/mm/yyyy): ___/___/___ to ___/___/___ |
| Have you travelled internationally within the last 14 days? | □ Yes □ No □ Unknown |
|  | If Yes, please specify:  Name of country visited  Name of cities visited in this country with dates of travel: ___/___/___ to ___/___/___ |
| Occupation | 11= Government job  12= Private job  13= Daily wages worker  14= Self employed  15= Farmer  16= Does not work  17= Other  99=Not applicable |

| **5. Household information** |  |
| --- | --- |
| GIS Location of household/Address of contact person if different from address of primary case |  |
| Date of last contact with the confirmed case (dd/mm/yyyy) | ___/___/___ |
| Does the contact share a room (or usually share a room) with the primary case? | □ Yes □ No □ Unknown |
| Number of days during the time the case was ill at home that were spent in contact with case |  |
| Did the contact take care of the case during the time he/she was ill at home? | □ Yes □ No □ Unknown |
| Did the contact hug the case during the time he/she was ill at home? | □ Yes □ No □ Unknown |
| Did the contact kiss the case during the time he/she was ill at home? | □ Yes □ No □ Unknown |
| Did the contact shake hands with the case during the time he/she was ill ? | □ Yes □ No □ Unknown |
| Did the contact share a meal with the case during the time he/she was ill? | □ Yes □ No □ Unknown |
| Did the contact eat with hands from the same plate as the case during the time he/she was ill? | □ Yes □ No □ Unknown |
| Did the contact share a drinking cup/glass with the case during the time he/she was ill? | □ Yes □ No □ Unknown |
| Did the contact share utensils with the case during the time he/she was ill ? | □ Yes □ No □ Unknown |
| Did the contact sleep in the same room as the case during the time he/she was ill? | □ Yes □ No □ Unknown |
| Did the contact share a toilet with the case during the time he/she was ill ? | □ Yes □ No □ Unknown |

| **6a. Symptoms in contact** |  |
| --- | --- |
| Has the contact experienced any respiratory symptoms (sore throat, runny nose, cough, shortness of breath) in the period from 4 days **before** symptom onset in the confirmed case until the present? | □ Yes □ No □ Unknown |
| Has the contact experienced any respiratory symptoms (sore throat, runny nose, cough, shortness of breath) in the period up to 14 days **after** the last contact or until the present date, whichever is the earlier? | □ Yes □ No □ Unknown |
| **6b. Currently ill** | □ Yes □ No □ Unknown |
| Date and time of first symptom onset (dd/mm/yyyy) | ___/___/___  □ am □ pm  □ Not applicable □ Unknown |
| Fever (≥38 °C) or history of fever | □ Yes □ No □ Unknown |
|  | If yes, date ___/___/___ |
|  | If Yes, specify maximum temperature:  ____°C □ Not applicable (na) |
| **6b. Respiratory symptoms** |  |
| Sore throat | □ Yes □ No □ Unknown  If yes, date ___/___/___ |
| Runny nose | □ Yes □ No □ Unknown  If yes, date ___/___/___ |
| Shortness of breath | □ Yes □ No □ Unknown  If yes, date ___/___/___ |
| Cough | □ Yes □ No □ Unknown  If yes, date ___/___/___ |
| **6c. Other symptoms** |  |
| Chills | □ Yes □ No □ Unknown |
| Vomiting | □ Yes □ No □ Unknown |
| Nausea | □ Yes □ No □ Unknown |
| Diarrhoea | □ Yes □ No □ Unknown |
| Headache | □ Yes □ No □ Unknown |
| Rash | □ Yes □ No □ Unknown |
| Conjunctivitis | □ Yes □ No □ Unknown |
| Muscle aches | □ Yes □ No □ Unknown |
| Joint ache | □ Yes □ No □ Unknown |
| Loss of appetite | □ Yes □ No □ Unknown |
| Loss of smell (anosmia) or taste | □ Yes □ No □ Unknown |
| Nose bleed | □ Yes □ No □ Unknown |
| Fatigue | □ Yes □ No □ Unknown |
| Seizures | □ Yes □ No □ Unknown |
| Altered consciousness | □ Yes □ No □ Unknown |
| Other symptoms | □ Yes □ No □ Unknown  If Yes, specify: |

| **7. Outcome (Day 1)** |  |
| --- | --- |
| Outcome | □ Alive □ Dead □ na □ Unknown |
| Outcome current as of date (dd/mm/yyyy) | ___/___/___  □ Unknown □ na |
| Hospitalization | □ Yes □ No □ Unknown |
|  | If Yes, date of first hospitalization (dd/mm/yyyy) |
|  | ___/___/___ |
|  | □ Unknown |
|  | If Yes, specify reason for hospitalization: |
|  |  |
| **8. Contact pre-existing condition(s)** |  |
| Pregnancy | □ Yes □ No □ Unknown |
|  | If Yes, specify trimester: |
|  | □ First □ Second □ Third □ Unknown |
| Obesity | □ Yes □ No □ Unknown |
| Cancer | □ Yes □ No □ Unknown |
| Diabetes | □ Yes □ No □ Unknown |
| HIV/other immune deficiency | □ Yes □ No □ Unknown |
| Heart disease | □ Yes □ No □ Unknown |
| Asthma (requiring medication) | □ Yes □ No □ Unknown |
| Chronic lung disease (non-asthma) | □ Yes □ No □ Unknown |
| Chronic liver disease | □ Yes □ No □ Unknown |
| Chronic haematological disorder | □ Yes □ No □ Unknown |
|  |  |
| Chronic kidney disease | □ Yes □ No □ Unknown |
| Chronic neurological impairment/disease | □ Yes □ No □ Unknown |
| Organ or bone marrow recipient | □ Yes □ No □ Unknown |
| Other pre-existing condition(s) | □ Yes □ No □ Unknown |
|  | If Yes, specify: |

| **10. Use of Mask** |  |
| --- | --- |
| Do you wear a face mask every time you go out? | 󠇫 Yes 󠇫always 󠇫 Wear it Sometimes 󠇫 󠇫 Never worn a mask |
|  |  |
| Type of mask you usually wear | 󠇫 S**urgical mask**  How often do you use surgical masks?   - Single time - More than once   󠇫 **Cloth mask**  How often do you wash masks?   - ____ times/day - ____ times/week - ____ times/month   󠇫 **Respirator** 󠇫 **Other**, specify ___________________ |
|  |  |
| **11. Lab sample collection** |  |
| Blood sample has been collected | Yes □ No □ |
|  |  |
| Nasal swab sample has been collected | Yes □ No □ |
|  | If yes to any of the two above,  Date of collection of samples: DD/MM/YY  Laboratory identification number: ______________ |
| **12. Status of form completion** |  |
| Form completed | □ Yes □ No □ partially filled |
|  | Specify reason only if No or partially filled: |
|  | □ Missed |
|  | □ Not attempted |
|  | □ Not performed |
|  | □ Refusal |
|  | □ Other, specify: |

***Form 2: Follow-up reporting form – for confirmed cases and household contacts (Day 7,14 and 28)***

**Unique Primary Case ID/Household number**

**Household Contact ID Number (C…):**

| 1**.Data Collector Information** |  |
| --- | --- |
| Name/code of data collector |  |
| Data Collector Phone Number |  |
| Visit date |  |
| Visit day (please check one) | □ Day 7  □ Day 14  □ Day 28 |
| **2. Interview respondent information (if different from initial visits)** | |
| First name |  |
| Family Name |  |
| Gender | □ Male □ Female |
| Date of birth (dd/mm/yyyy) | ___/___/___ □ Unknown |
|  |  |
| Relationship to participant |  |
|  |  |
| Respondent address/GIC coordinates |  |
|  |  |
| Telephone (mobile) number |  |

| **3a. Respiratory symptoms** | |
| --- | --- |
| Fever | □ Yes □ No □ Unknown  Date of onset: __/__/__ |
| Maximum temperature (specify) | __°C □ Not applicable (na) |
| **3b. Respiratory symptoms** |  |
| Sore throat | □ Yes □ No □ Unknown |
|  | If Yes, date (dd/mm/yyyy) ___/___/___ |
| Runny nose | □ Yes □ No □ Unknown |
| Cough | □ Yes □ No □ Unknown |
|  | If Yes, date (dd/mm/yyyy) ___/___/___ |
| Shortness of breath | □ Yes □ No □ Unknown |
|  | If Yes, date (dd/mm/yyyy) ___/___/___ |
| **3c. Other symptoms** |  |
| Chills | □ Yes □ No □ Unknown |
| Vomiting | □ Yes □ No □ Unknown |
| Nausea | □ Yes □ No □ Unknown |
| Diarrhoea | □ Yes □ No □ Unknown |
| Headache | □ Yes □ No □ Unknown |
| Rash | □ Yes □ No □ Unknown |
| Conjunctivitis | □ Yes □ No □ Unknown |
| Muscle aches | □ Yes □ No □ Unknown |
| Joint ache | □ Yes □ No □ Unknown |
| Loss of appetite | □ Yes □ No □ Unknown |
| Nose bleed | □ Yes □ No □ Unknown |
| Fatigue | □ Yes □ No □ Unknown |
| Seizures | □ Yes □ No □ Unknown |
| Altered consciousness | □ Yes □ No □ Unknown |
| Other neurological signs | □ Yes □ No □ Unknown |
|  | If Yes, specify |
| Other symptoms | □ Yes □ No □ Unknown |
|  | If Yes, specify: |

| **4. Pre-existing condition(s)** |  |
| --- | --- |
| Pregnancy | □ Yes □ No □ Unknown |
|  | If Yes, specify trimester:  □ First □ Second □ Third □ Unknown  If No,   1. Not pregnant at the time of enrollment 2. Miscarriage/ Abortion 3. Still birth 4. Delivered |
| **5. Complications** | |
| Hospitalization | □ Yes □ No □ Unknown |
| Date of first hospitalization (dd/mm/yyyy) | ___/___/___ |
|  | □ Unknown |
| ICU (intensive care unit) admission | □ Yes □ No □ Unknown |
| ICU admission | ___/___/___ |
|  | □ Unknown |
| Date of discharge from ICU (dd/mm/yyyy) | ___/___/___ |
|  | □ Unknown □ na |
| Mechanical ventilation | □ Yes □ No □ Unknown |
|  |  |
| Dates of mechanical ventilation (dd/mm/yyyy) | Start___/___/___ |
|  | Stop___/___/___ |
|  | □ Unknown □ na |
| Length of ventilation (days) |  |
| Acute respiratory distress syndrome (ARDS) | □ Yes □ No □ Unknown |
|  | If Yes, date started (dd/mm/yyyy)___/___/___ |
| Acute renal failure | □ Yes □ No □ Unknown |
|  | If Yes, date started (dd/mm/yyyy)___/___/___ |
| Cardiac failure | □ Yes □ No □ Unknown |
|  | If Yes, date started (dd/mm/yyyy)___/___/___ |
| Consumptive coagulopathy | □ Yes □ No □ Unknown |
|  | If Yes, date started (dd/mm/yyyy)___/___/___ |
| Pneumonia by chest X-ray | □ Yes □ No □ Unknown |
|  | If Yes, date started (dd/mm/yyyy)___/___/___ |
| Other complications | □ Yes □ No □ Unknown |
|  | If Yes, specify: |
| Hypotension requiring vasopressors | □ Yes □ No □ Unknown |
| Extracorporeal membrane oxygenation (EMO) required | □ Yes □ No □ Unknown |

| **6. Secondary bacterial infection**  Complete a new line for each specimen collected and each type of test done (if applicable) | | |
| --- | --- | --- |
| Date of sample (dd/mm/yyyy) | Type of sample | Positive results |
|  | □ Sputum | □ Haemophilus influenza |
|  | □ Endotracheal aspirate | □ MRSA |
|  | □ Pleural fluid | □ Staphylococcus aureus |
|  | □ CSF | □ Streptococcus pneumoniae |
|  | □ Blood | □ E. coli |
|  | □ Urine | □ Other organism, please specify: |
|  | □ Faeces |  |
|  | □ Other, please specify: |  |
| **8. Final contact classification (at final follow-up day 28 for contacts only)** |  | |
| Please mark | □ Never ill/not a case | |
|  | □ Confirmed secondary case | |
|  | □ Lost to follow-up | |
|  | □ Suspected case | |
|  | □ Probable case | |
| **9.Outcome** |  | |
| Outcome | □ Alive □ Dead □ na □ Unknown | |
|  | Recovered: □ Yes □No □Unknown  if Yes, specify date symptoms resolved  (dd/mm/yyyy) __/___/___ | |
|  | Still ill | |
|  | If Dead, specify date of death (dd/mm/yyyy)  ­­___/___/___ | |
|  | Unknown, lost to follow-up | |

| **10. Use of Mask** |  |
| --- | --- |
| Do you wear a face mask every time you go out? | 󠇫 Yes 󠇫always 󠇫 Wear it Sometimes 󠇫 󠇫 Never worn a mask |
|  |  |
| Type of mask you usually wear | 󠇫 S**urgical mask**  How often do you use surgical masks?   - Single time - More than once   󠇫 **Cloth mask**  How often do you wash masks?   - ____ times/day - ____ times/week - ____ times/month   󠇫 **Respirator** 󠇫 **Other**, specify ___________________ |
|  |  |

| **11. Lab sample collection** |  |
| --- | --- |
| Blood sample has been collected | Yes □ No □ |
|  |  |
| Nasal swab sample has been collected | Yes □ No □ |
|  | If yes to any of the two above,  Date of collection of samples: DD/MM/YY  Laboratory identification number: ______________ |

| **12. Status of form completion** |  |
| --- | --- |
| Form completed | □ Yes □ No □ partially filled |
|  | Specify reason only if No or partially filled: |
|  | □ Missed |
|  | □ Not attempted |
|  | □ Not performed |
|  | □ Refusal |
|  | □ Other, specify: |

*Form 3: Lab results reporting form– for confirmed cases and household contacts (Day 1, 7, 14, 28)*

This table will need to be completed for every specimen collection at each point at the basilne and in the follow-up for case and households contact, depending on the chosen specimen-collection schedule.

**1a. Virology testing methods and results:**

**Complete a new line for each specimen collected and each type of test done:**

| **Laboratory** | **Date sample** | **Date sample** |  |  |  |  |  |  |  |  | **Specimens shipped** |
| --- | --- | --- | --- | --- | --- | --- | --- | --- | --- | --- | --- |
| **identification** | **collected** | **received** |  |  |  |  |  | **Result date** | |  | **to other laboratory** |
| **number** | **(dd/mm/yyyy)** | **(dd/mm/yyyy)** | **Type of sample** | | **Type of test** | | **Result** | **(dd/mm/yyyy)** | |  | **for confirmation** |
|  | ___/___/___ | ___/___/___ | □ Nasal swab | | □ PCR | | □ POSITIVE for COVID-19 | ___/___/___ |  |  | □ Yes |
|  |  |  | □ Throat swab | | □ Whole genome | |  |  |  |  | If Yes, specify date |
|  |  |  | □ | | sequencing | | □ NEGATIVE for COVID-19 |  |  |  | ___/___/___ |
|  |  |  | Nasopharyngeal | | □ Partial genome | |  |  |  |  | If Yes, name of the |
|  |  |  | swab | | sequencing | | □ POSITIVE for other |  |  |  | laboratory: |
|  |  |  | □ Other, specify: | | □ Other, specify | | pathogens |  |  |  |  |
|  |  |  |  |  |  |  | Please specify which |  |  |  | □ No |
|  |  |  |  |  |  |  | pathogens: |  |  |  |  |
|  |  |  |  |  |  |  |  |  |  |  |  |
|  |  |  |  |  |  |  |  |  |  |  |  |
| **1b. Serology testing methods and results:** | | |  |  |  |  |  |  |  |  |  |
| **Complete a new line for each specimen collected and each type of test done:** | | | | | | |  |  |  |  |  |
| **Laboratory** | **Date sample** | **Date sample** | **Type of** |  | **Type of test** |  | **Result (COVID-19** | **Result date** |  | **Specimens shipped** | |
| **identification** | **collected** | **received** | **sample** |  |  |  | **antibody titres)** | **(dd/mm/yyyy)** |  | **to other laboratory** | |
| **number** | **(dd/mm/yyyy)** | **(dd/mm/yyyy)** |  |  |  |  |  |  |  |  | **for confirmation** |
|  | ___/___/___ | ___/___/___ | □ Serum |  | Specify type |  | □ POSITIVE | ___/___/___ |  | □ Yes | |
|  |  |  | □ Other, |  | (ELISA/IFA IgM/IgG, |  | If positive, titre: |  |  | If Yes, specify date | |
|  |  |  | specify: |  | neutralization assay, |  |  |  |  | ___/___/___ | |
|  |  |  |  |  | etc.): |  | □ NEGATIVE |  |  | If Yes, name of the | |
|  |  |  |  |  |  |  | □ INCONCLUSIVE |  |  | laboratory: ____ | |
|  |  |  |  |  |  |  |  |  |  | □ No | |
|  |  |  |  |  |  |  |  |  |  |  |  |

**Household Transmission Dynamics of COVID-19 In Karachi, Pakistan**

**Symptom Diary**

**Name of the Participant:**_______________________________________ **Participant ID:** _______________________________________

**Household ID:** _______________________________________

**Name of study staff completing this checklist:** _______________________________________ |___|___|___|

*You should put ‘√’ mark in the appropriate boxes.*]

| **Day & Date**  **(Day 1-28)** | **Do you have any of the following symptoms developed within last 24 hours?** | | | | | | | | | | | | | | | | | | | | | | | |  |
| --- | --- | --- | --- | --- | --- | --- | --- | --- | --- | --- | --- | --- | --- | --- | --- | --- | --- | --- | --- | --- | --- | --- | --- | --- | --- |
|  | **Fever or chills** | | **Sore throat** | | **Congestion or runny nose** | | **Cough** | | **Shortness of breath/**  **Difficulty breathing** | | **Nausea or vomiting** | | **Diarrhea** | | **Headache** | | **muscle/**  **body aches** | | **New loss of smell** | | **New loss of taste** | | **New fatigue** | | **Any other symptoms?** |
|  | **Y** | **N** | **Y** | **N** | **Y** | **N** | **Y** | **N** | **Y** | **N** | **Y** | **N** | **Y** | **N** | **Y** | **N** | **Y** | **N** | **Y** | **N** | **Y** | **N** | **Y** | **N** |  |
| __ /__ /__ |  |  |  |  |  |  |  |  |  |  |  |  |  |  |  |  |  |  |  |  |  |  |  |  |  |
| __ /__ /__ |  |  |  |  |  |  |  |  |  |  |  |  |  |  |  |  |  |  |  |  |  |  |  |  |  |
| __ /__ /__ |  |  |  |  |  |  |  |  |  |  |  |  |  |  |  |  |  |  |  |  |  |  |  |  |  |
| __ /__ /__ |  |  |  |  |  |  |  |  |  |  |  |  |  |  |  |  |  |  |  |  |  |  |  |  |  |
| __ /__ /__ |  |  |  |  |  |  |  |  |  |  |  |  |  |  |  |  |  |  |  |  |  |  |  |  |  |
| __ /__ /__ |  |  |  |  |  |  |  |  |  |  |  |  |  |  |  |  |  |  |  |  |  |  |  |  |  |
| __ /__ /__ |  |  |  |  |  |  |  |  |  |  |  |  |  |  |  |  |  |  |  |  |  |  |  |  |  |
| __ /__ /__ |  |  |  |  |  |  |  |  |  |  |  |  |  |  |  |  |  |  |  |  |  |  |  |  |  |
| __ /__ /__ |  |  |  |  |  |  |  |  |  |  |  |  |  |  |  |  |  |  |  |  |  |  |  |  |  |
| __ /__ /__ |  |  |  |  |  |  |  |  |  |  |  |  |  |  |  |  |  |  |  |  |  |  |  |  |  |
| __ /__ /__ |  |  |  |  |  |  |  |  |  |  |  |  |  |  |  |  |  |  |  |  |  |  |  |  |  |
| __ /__ /__ |  |  |  |  |  |  |  |  |  |  |  |  |  |  |  |  |  |  |  |  |  |  |  |  |  |
| __ /__ /__ |  |  |  |  |  |  |  |  |  |  |  |  |  |  |  |  |  |  |  |  |  |  |  |  |  |
| __ /__ /__ |  |  |  |  |  |  |  |  |  |  |  |  |  |  |  |  |  |  |  |  |  |  |  |  |  |
| __ /__ /__ |  |  |  |  |  |  |  |  |  |  |  |  |  |  |  |  |  |  |  |  |  |  |  |  |  |
| __ /__ /__ |  |  |  |  |  |  |  |  |  |  |  |  |  |  |  |  |  |  |  |  |  |  |  |  |  |
| __ /__ /__ |  |  |  |  |  |  |  |  |  |  |  |  |  |  |  |  |  |  |  |  |  |  |  |  |  |
| __ /__ /__ |  |  |  |  |  |  |  |  |  |  |  |  |  |  |  |  |  |  |  |  |  |  |  |  |  |
| __ /__ /__ |  |  |  |  |  |  |  |  |  |  |  |  |  |  |  |  |  |  |  |  |  |  |  |  |  |

# **COVID-19 Transmission Dynamics Survey in Karachi**

# **Close contact Tracing Worksheet**

When interviewing a case to identify potential close contacts, consider **all individuals that could have had exposure since the case was symptomatic and 4 days prior to the first onset of symptoms**.

Use the following activity prompts to help identify potential close contacts: work; school; visitors at home; volunteer activities; daycare; religious activities; social activities (restaurants, shopping); sports; visits to acute care settings, long-term care homes, retirement homes, medical labs, dentists, and other health care providers; contact with ill persons; and contact with birds or other animals.

| Today’s date | DD/MM/YY |
| --- | --- |
| Date of diagnosis | DD/MM/YY |
| Date of symptom onset | DD/MM/YY |
| Date of birth | DD/MM/YY |
| Gender | 1. Female 2. Male |

| Date/Time (Start and End) | Activities | Location of Activity | Name & contact information of potential close contacts | ADDRESS | PHONE NUMBER | Comments |
| --- | --- | --- | --- | --- | --- | --- |
|  |  |  |  |  |  |  |
|  |  |  |  |  |  |  |
|  |  |  |  |  |  |  |
|  |  |  |  |  |  |  |
|  |  |  |  |  |  |  |
|  |  |  |  |  |  |  |
|  |  |  |  |  |  |  |
|  |  |  |  |  |  |  |
|  |  |  |  |  |  |  |
|  |  |  |  |  |  |  |
|  |  |  |  |  |  |  |
|  |  |  |  |  |  |  |
|  |  |  |  |  |  |  |
|  |  |  |  |  |  |  |
|  |  |  |  |  |  |  |
|  |  |  |  |  |  |  |
|  |  |  |  |  |  |  |
